# Rational Design of Hollow Nanostructures: Engineering the Cavity Microenvironment for Advanced Electrocatalysis

**DOI:** 10.3390/nano16060360

**Published:** 2026-03-15

**Authors:** Yong-Gang Sun, Xin Wang, Jian Xiong, Yi-Han Zhang, Jin-Yi Ding, Bo Peng, Yuan Gu, Yi-Cong Xie, Kang-Lin Zhang, Mao Yuan, Xi-Jie Lin

**Affiliations:** 1 School of Chemistry & Chemical Engineering, Yancheng Institute of Technology, Yancheng 224051, China; wang92643864@163.com (X.W.); xiongjian1917@163.com (J.X.); ruarua0505@163.com (Y.-H.Z.); djy11062026@163.com (J.-Y.D.); pengbo5119@163.com (B.P.); gunyuangygy@163.com (Y.G.); xie20050701@outlook.com (Y.-C.X.); kanglin857@gmail.com (K.-L.Z.); pzznjb@163.com (M.Y.); 2 School of Chemistry and Pharmaceutical Sciences, Guangxi Normal University, Guilin 541004, China

**Keywords:** hollow nanostructures, cavity microenvironment, electrocatalysis, rational design, structure–property relationships

## Abstract

Hollow nanostructures have emerged as a pivotal class of nanomaterials in electrocatalysis, offering intrinsic advantages such as high surface-to-volume ratios, reduced density, and economical utilization of precious metals. However, the prevailing research paradigm has predominantly focused on the external shell characteristics while overlooking the decisive role of the interior cavity microenvironment. This review introduces a novel conceptual framework that positions the rational engineering of the cavity microenvironment—encompassing mass transport dynamics, localized electronic structure modulation, active site exposure, and structural stability—as a unified design principle for next-generation electrocatalysts. We systematically elucidate how precise control over cavity geometry, composition, and interfacial properties can optimize electrocatalytic performance for oxygen reduction (ORR), oxygen evolution (OER), and hydrogen evolution (HER) reactions. By correlating microenvironmental parameters with catalytic metrics, we establish structure–property–performance relationships and highlight recent breakthroughs. Finally, we outline future challenges in achieving atomic-level precision in cavity design, understanding dynamic evolution under operating conditions, and scaling up synthesis for industrial applications.

## 1. Introduction

In the global transition towards sustainable energy systems, efficient electrochemical technologies such as fuel cells [1,2], water electrolyzers [3,4], and metal–air batteries [5,6] have become critical supports and indispensable components. At the core of these devices are three key electrocatalytic reactions: the oxygen reduction reaction (ORR), the oxygen evolution reaction (OER), and the hydrogen evolution reaction (HER) [7,8,9]. Their efficiency directly determines the performance of the entire energy conversion process. However, the limitations of existing electrocatalysts have hindered the large-scale commercial application of these technologies [10,11]. Researchers have long struggled to balance high catalytic activity, long-term stability under harsh operating conditions, and cost-effectiveness [12,13]. This challenge is particularly exacerbated by reactions involving multi-step proton–electron transfers, such as ORR and OER, which inherently proceed at slow reaction rates [14,15]. Developing advanced catalyst structures that can simultaneously address these interrelated issues has therefore become an important focus in the current scientific research and engineering fields.

Against this backdrop, hollow nanostructures have emerged as a research hotspot in catalyst design due to their unique structural characteristics [16,17]. Conventional studies generally recognize the inherent advantages of such structures: the large specific surface area of hollow structures can increase the density of active sites per unit mass, directly enhancing catalytic efficiency [18,19]. For example, the specific surface area of hollow mesoporous SiO_2_ materials can reach 224 m^2^/g [20], much higher than that of similar solid materials, enabling them to provide more active sites for catalytic reactions. Meanwhile, their high porosity and high surface area not only allow the electrolyte to form a uniform interface with the catalyst but also help the electrolyte penetrate deeper into the catalyst layer, reducing mass transfer resistance at the electrode–electrolyte interface. Taking common precious metal catalysts such as platinum and iridium as examples, hollow structures can maintain or even improve catalytic performance while reducing material usage, significantly enhancing cost-effectiveness. Studies have shown that Pt/Ag hollow nanostructures can achieve desirable performance with only a low precious metal loading, exhibiting a mass activity of 0.438 A mg_Pt_^−1^ at 0.9 V (vs. RHE), which is approximately three times higher than that of commercial Pt/C toward the oxygen reduction reaction [21,22]. In addition, the shell porosity of hollow structures is usually tunable, which allows reactants to easily access the internal active sites while facilitating the timely desorption of products such as oxygen and hydrogen bubbles, as well as reaction intermediates [23,24]. This is crucial for maintaining long-term catalytic stability and avoiding catalyst poisoning. Collectively, these characteristics make hollow nanostructures an effective solution to the three interrelated challenges of activity, stability, and cost in electrocatalysis.

But have these advantages been fully exploited? There is a critical research gap in the current field: the internal cavity of hollow structures is mostly treated as a passive void rather than an engineerable active region [25,26,27]. This oversight ignores the profound impact of the cavity microenvironment—a confined space bounded by the inner surface of the shell—on the catalytic process, severely limiting the full potential of hollow nanostructures. We therefore argue that a paradigm shift is needed: the cavity microenvironment should be regarded as a key design parameter. This engineerable space actively influences several critical aspects: first, mass transport properties. The confined volume regulates diffusion rates and forms local concentration gradients of reactants and products, which may either promote or hinder reaction kinetics [28,29]. For instance, the Au@Cu_2_O yolk-shell nanoreactors can alter the permeability of CO_2_ by adjusting the cavity size [30], thereby regulating the reaction pathway. Second, the local electronic environment. Curvature-induced charge redistribution in the shell, combined with shell composition and quantum confinement effects, can change the electronic states of reaction intermediates [31,32]. Third, the accessibility and stability of active sites. The cavity structure can protect active sites from aggregation and leaching while providing additional catalytic surfaces that are inaccessible in solid structures [33,34]. Fourth, mechanical stress distribution and strain engineering. The inherent curvature generates lattice strain that can adjust the electronic structure of active sites, optimizing the adsorption energy of key intermediates [35,36]. Clearly, the systematic engineering of these microenvironmental factors, rather than merely utilizing the hollow morphology, can open up new directions for the development of advanced electrocatalysts.

Based on this perspective, this review proposes a conceptual framework: the rational design and regulation of the cavity microenvironment—including mass transport dynamics, local electronic structure, active site exposure, and structural stability—as the core design principle for next-generation electrocatalysts. We will systematically illustrate how precise control over the geometric shape, composition, and interfacial properties of the cavity can optimize its performance in ORR, OER, and HER. Meanwhile, we will summarize the advanced theoretical methods essential for deciphering the cavity microenvironment, and finally prospect the current challenges and future opportunities in this field, including atomic-level precise design, understanding of dynamic processes under operating conditions, and scalable synthesis.

## 2. Engineering the Cavity Microenvironment: A Design Principle

The catalytic performance of hollow nanostructures is highly dependent on the intrinsic properties of their cavity microenvironment, and the precise engineering regulation of this microenvironment has become a core design criterion for developing next-generation high-efficiency electrocatalysts. The core characteristics of the cavity microenvironment are collectively determined by geometric structural parameters, chemical composition, interfacial properties, and dynamic reaction processes. These factors ultimately dominate the kinetic processes and thermodynamic limits of electrocatalytic reactions by regulating mass transport efficiency, electronic structure arrangement, active site exposure, and stability.

### 2.1. Geometric Dimensionality Control

The geometric parameters of the cavity are the basis for shaping the characteristics of the microenvironment, mainly including cavity diameter (D), shell thickness (t), chamber configuration (single-chamber/multi-chamber), and hierarchical porous structure. These parameters do not act in isolation but indirectly affect the rate and selectivity of catalytic reactions by synergistically regulating mass transport efficiency, electronic conductivity, mechanical stability, and spatial confinement effects [18,37]. Clarifying the physicochemical effects of each geometric parameter and their electrocatalytic response laws is a prerequisite for achieving precise regulation of the cavity microenvironment. Table 1 systematically summarizes the core impacts of key geometric parameters and the corresponding electrocatalytic application orientations, providing quantitative references for the rational design of geometric structures.

In the synergistic regulation of geometric parameters for hollow-structured catalysts, shell thickness and cavity size are the key factors determining the balance between mass transport efficiency and structural stability [47,48]. For reactions such as ORR that require rapid gas diffusion to maintain high limiting current density, a thin shell combined with a large cavity is beneficial to minimize diffusion resistance, while ensuring the shell has sufficient mechanical stability. Conversely, for catalysts operating under harsh oxidative conditions (high potential, alkaline electrolyte), such as OER, a relatively thicker shell is more advantageous [49,50]. The increased shell thickness effectively enhances structural robustness, thereby inhibiting structural degradation during long-term operation. Research has confirmed that precise tuning of the geometric parameters of hollow structures can significantly optimize catalytic performance. A study on hollow carbon spheres (HCS) demonstrated that tuning the particle size, which inherently changes the cavity dimension and shell thickness, directly impacts ORR activity [51]. HCS with a diameter of 200 nm, possessing an ultrathin carbon shell (~20 nm), exhibited superior ORR performance, attributed to its high surface area and favorable structure for reactant diffusion. Yang et al. [52] investigate the synthesis of nitrogen-doped mesoporous hollow carbon spheres (N-MHCS) with tunable particle sizes and their application as non-precious metal catalysts for ORR in fuel cells. They demonstrate that engineering the particle size of hollow carbon spheres is a critical strategy for optimizing ORR catalysis. The ~150 nm N-MHCS achieved an ideal synergy of high active site density, efficient mass transport, and conductive pathways, showcasing great potential as a cost-effective and durable alternative to platinum-based catalysts.

**Figure 1:**
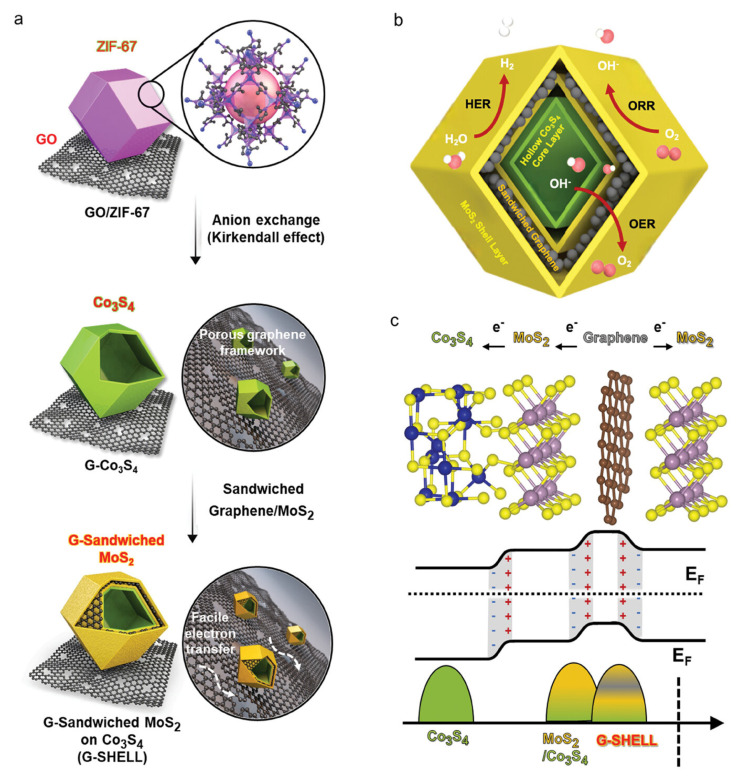
Schematics of the trifunctional graphene-sandwiched heterojunction-embedded layered lattice (G-SHELL): (**a**) its synthesis from a zeolitic imidazole framework, (**b**) the hollow core-layered shell structure design with active sites for ORR, OER, and HER, and (**c**) the heterojunctions, induced internal electric fields, and corresponding electronic band structure [54].

Furthermore, structural designs featuring multiple cavities or multi-shell architectures have demonstrated significant advantages in catalysis [24,53]. A prime example is the trifunctional graphene-sandwiched heterojunction-embedded layered lattice (G-SHELL) electrocatalyst, which embodies this design principle [54]. The synthesis procedure, depicted in Figure 1a, involves a solvothermal route where graphene oxide (GO) serves as a template for sequential ZIF-67 growth, sulfidization, and MoS_2_ deposition, ultimately forming a hollow Co_3_S_4_ core surrounded by a layered MoS_2_ shell encapsulated within conductive graphene sheets. This sophisticated multi-shell configuration, illustrated in Figure 1b, creates confined nanospaces that significantly enhance ion transport to both the inner Co_3_S_4_ layer (with OER sites) and the outer MoS_2_ layers (with ORR/HER sites), while the hollow morphology increases active site accessibility and reduces steric hindrance. The electronic structure of G-SHELL is further elucidated in Figure 1c. As shown in Figure 1c, the sandwiched graphene layer donates electrons to the van der Waals-bonded MoS_2_ layer, which subsequently transfers electrons to the Co_3_S_4_ layer. This electron transfer pathway is confirmed by the calculated electronic band structure presented in Figure 1c, which also reveals the formation of heterojunction-induced internal electric fields (IEFs). These IEFs dramatically accelerate electron migration to active sites for OER, ORR, and HER, promoting rapid redox kinetics and high activity. The synergistic effects arising from this integrated design endow the catalyst with exceptional activity for all three reactions. The practical efficacy of this multi-shell strategy is convincingly validated by the high-performance, integrated system where the G-SHELL catalyst enables a Zn-air battery to efficiently drive overall water splitting, achieving remarkable power density and operational stability.

Similarly, the introduction of hierarchically porous structures within catalyst frameworks offers another powerful dimension for performance optimization, primarily by simultaneously maximizing the exposure of active sites and ensuring efficient mass transport. Specifically, the hierarchical pore structure possesses abundant accessible internal voids, high specific surface area, and interconnected multiscale pores (micro/meso/macropores), which provide sufficient and stable anchoring sites for metal species. These pores can accommodate and disperse metal nanoparticles/precursors within the hollow interior and pore channels, effectively suppressing aggregation. Consequently, the hierarchical structure enables a higher metal mass loading while maintaining good dispersion and utilization efficiency of metal sites. Cui et al. [55] employed a soft-template combined with a bidentate chelation strategy to fabricate transition metal single-atom catalysts (TM-SAC-HC) featuring hollow nanospheres with hierarchically porous carbon shells. This specific hierarchical pore structure, integrating different pore sizes, was crucial for achieving an ultra-high metal loading of up to 9.36 wt.% while effectively preventing the aggregation or burial of single-atom sites. The interconnected pore network facilitated the rapid diffusion of reactants and electrolytes, which, combined with the high density of accessible active sites, led to exceptional ORR performance.

### 2.2. Chemical Composition and Interfacial Design

Geometric parameters provide a basic structural framework for the cavity microenvironment, while precise regulation of chemical composition and interfacial properties further endows it with customizable electronic characteristics and surface functions. By regulating the shell chemical composition, constructing interfacial heterostructures, and modifying surface hydrophilicity/hydrophobicity, the electronic structure of active sites, the adsorption energy of reaction intermediates, and the interfacial interaction between reactants/products can be precisely adjusted, thereby breaking through the performance bottleneck of traditional geometric regulation.

#### 2.2.1. Shell Doping and Confinement Effects

**Figure 2:**
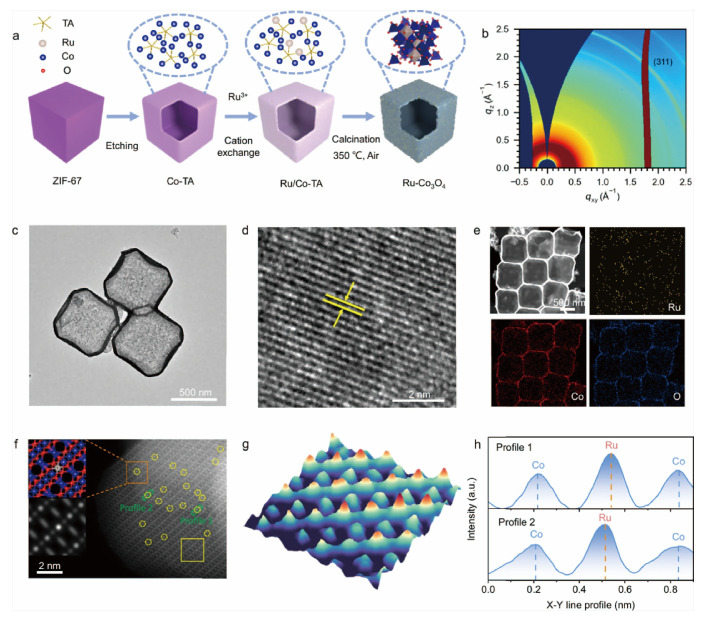
Schematic illustration and structural characterization of Ru single-atom decorated Co_3_O_4_ (Ru-Co_3_O_4_). (**a**) The synthesis process. (**b**) Wide-angle X-ray scattering (WAXS) pattern. (**c**,**d**) TEM and HR-TEM images. (**e**) HAADF-STEM image and corresponding elemental maps. (**f**,**g**) Atomic-resolution HAADF-STEM image and 3D Gaussian fitting confirming isolated Ru atoms (highlighted). (**h**) Intensity profile across the selected region [58].

Introducing heteroatoms (N, P, S, B, etc.) into the shell of hollow structures is a classic strategy for regulating the local electronic structure. Its core goal is to optimize the adsorption energy of reaction intermediates and improve catalytic activity and selectivity. Heteroatom doping mainly exerts its effect through three synergistic mechanisms: ligand effect, ensemble effect, and strain effect. The ligand effect originates from the electronegativity difference between heteroatoms and the host lattice. This fundamental difference profoundly alters the electronic structure of carbon matrices, which in turn governs their efficacy in reactions such as the oxygen reduction reaction [56]. For heteroatoms with higher electronegativity than carbon (χ = 2.55), such as nitrogen (χ = 3.04) and fluorine (χ = 4.0), doping induces a redistribution of charge and spin density on the adjacent sp^2^-hybridized carbon lattice. This creates electron-deficient sites and structural defects with high reactivity, which facilitate key steps like the initial electronic interaction with O_2_ molecules and the cleavage of the O=O bond, steering the pathway toward efficient 4-electron reduction. Conversely, doping with elements of lower electronegativity, like phosphorus (χ = 2.19), generates positively charged centers that also effectively promote the adsorption and activation of reactants. It should be clarified that this promotion effect is not universal across all electrocatalytic reactions: it is more prominent in reactions involving small-molecule reactants (e.g., O_2_, H_2_O) that depend on electron transfer and bond cleavage at active sites, while for reactions with large-molecule reactants or those limited by factors other than reactant adsorption/activation (e.g., mass transport, product desorption), this promotion may be weakened or negligible. Even for dopants with similar electronegativity, such as sulfur (χ = 2.58), catalytic performance can be enhanced through alternative mechanisms like significant enhancement of spin density. The ensemble effect refers to the fact that defect sites (e.g., vacancies, edge sites) formed during the doping process can serve as active centers or coordinate with metal atoms to form unique active site structures (e.g., M-N_x_, M-P_x_) [57]. Such sites are difficult to form in undoped samples and often have more excellent catalytic performance. The strain effect is caused by lattice distortion induced by heteroatom doping. Local lattice strain can adjust the d-band center position of transition metals, thereby optimizing the adsorption energy of intermediates. A representative example is the incorporation of larger Ru single atoms into the Co_3_O_4_ lattice, as illustrated in Figure 2 [58]. Hollow Co_3_O_4_ nanoboxes were synthesized using ZIF-67 nanocubes as templates via tannic acid-assisted chemical etching and thermochemical calcination, followed by cation exchange to introduce Ru single atoms, yielding Ru-Co_3_O_4_ nanobox catalysts (Figure 2a). Structural analyses and electron microscopy imaging confirm that the spinel framework of Co_3_O_4_ is largely preserved after Ru incorporation (Figure 2b–f). The incorporation of larger Ru single atoms into the Co_3_O_4_ lattice induced significant local compressive strain, leading to lattice distortion and a noticeable shortening of the Co–O bond lengths, further supported by 3D atom-overlapping Gaussian function fitting and intensity profiles (Figure 2g,h). This structural perturbation directly modulated the electronic environment around the active Co sites, effectively optimizing the adsorption strength of oxygenated intermediates. Consequently, the catalyst exhibited a remarkably low overpotential of 238 mV at 10 mA cm^−2^ and sustained operation for over 120 h in acidic media, showcasing how intentional strain engineering through heteroatom doping can simultaneously enhance both activity and stability in challenging electrochemical reactions.

**Figure 3:**
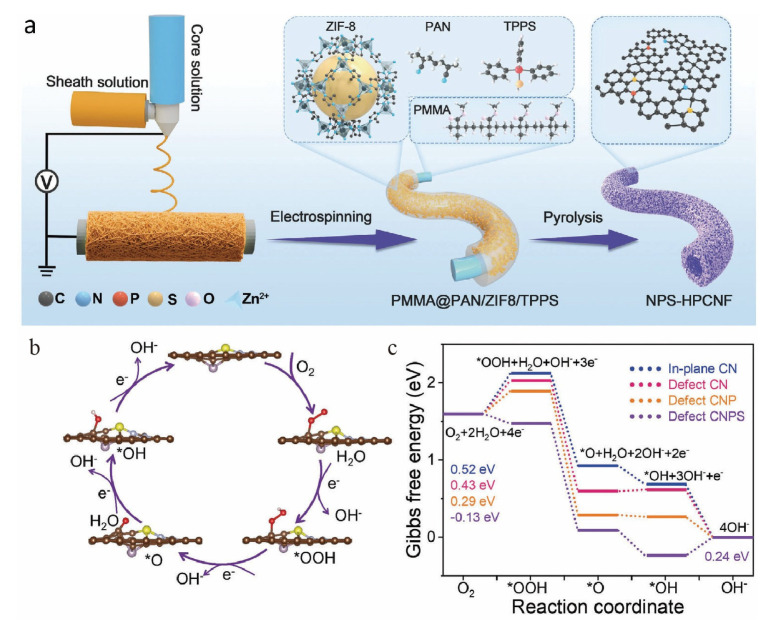
Preparation schematic, adsorption configuration, and ORR pathway analysis of NPS-HPCNF. (**a**) Schematic illustration of the NPS-HPCNF preparation process. (**b**) Optimized configurations of reaction intermediates adsorbed at the defect site of the CNPS model (atoms: C-brown, N-silver, P-purple, S-yellow, O-red). (**c**) Calculated free energy diagrams for ORR on four different models at the equilibrium potential [60].

Nitrogen doping is the most widely used doping strategy in carbon-based hollow catalysts, and its mechanism has been fully verified. A heteroatom-doping strategy is reported to precisely steer the ORR pathway on N-doped hollow mesoporous carbon [59,60]. Figure 3a schematically illustrates a fabrication process for N, P, and S tri-doped hollow carbon nanofibers. Doping with phosphorus generates electron-rich C–P domains, which cooperate with graphitic-N sites, promote *OOH desorption and selectively catalyze the 2e^−^ reduction to H_2_O_2_. Conversely, sulfur doping introduces electron-deficient C–S domains that strongly bind *OOH, cooperating with pyridinic-N sites to drive O–O bond cleavage and the 4e^−^ pathway to H_2_O. Gibbs free energy (ΔG) profiles for the 4e^−^ ORR pathway (Figure 3b,c) reveal that while in-plane CN, defect CN, and defect CNP models exhibit nonspontaneous *OOH formation as the rate-determining step (RDS) with ΔG values of 0.52, 0.43, and 0.29 eV, respectively, the defect CNPS model demonstrates spontaneous *OOH formation and a significantly lower RDS barrier of 0.24 eV for *OH reduction. The activity correlates positively with graphitic-N content, confirming its role as the primary active site for efficient electron transfer. Another carefully designed catalyst features Co, N-doped carbon nanotubes grown on nitrogen-doped hollow carbon polyhedrons [61]. The introduced nitrogen species, particularly in coordination with cobalt to form Co-N_x_ active sites, synergistically create an electron-rich environment. This optimized electronic structure not only facilitates the adsorption and stabilization of OOH intermediates but also concurrently promotes charge transfer, thereby significantly boosting the ORR activity. Consequently, the catalyst achieved a remarkable half-wave potential of 0.87 V vs. RHE for ORR. More importantly, the nitrogen-modulated electronic effects, combined with the unique hollow structure of nitrogen-doped carbon polyhedrons and grafted Co, N-doped carbon nanotube structure, also endowed the material with excellent OER and HER performance. The hollow structure affords a large surface area and fast mass transport, while the grafted carbon nanotubes enhance conductivity and structural stability. These enabled its successful application as an integrated electrode in both zinc-air batteries and self-powered overall water-splitting systems, demonstrating the critical role of nitrogen doping in constructing high-performance, multifunctional electrocatalysts.

**Figure 4:**
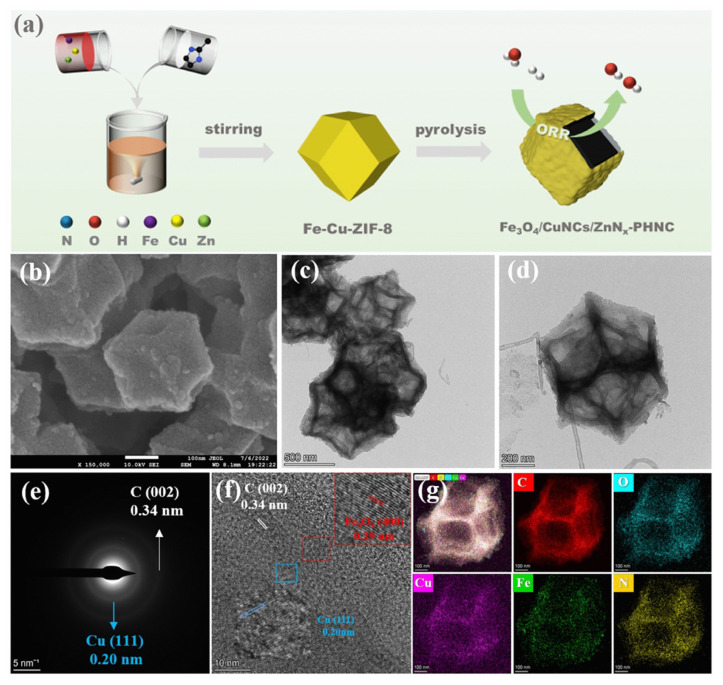
Synthesis and structural characterization of the Fe_3_O_4_/CuNCs/ZnN_x_-PHNC catalyst. (**a**) Schematic preparation flowchart; (**b**) SEM images; (**c**,**d**) TEM images at different magnifications; (**e**) SAED pattern; (**f**) HRTEM image; (**g**) HAADF-STEM image and corresponding elemental mapping [63].

It is worth noting that the synergistic effect of doping and cavity confinement environment may produce more significant catalytic enhancement effects. Researchers ingeniously pre-encapsulated iron phthalocyanine (FePc) molecules within the cavities of a ZIF-8 metal–organic framework [62]. During subsequent pyrolysis, the rigid cavity structure acted as a nanoscale reactor, providing spatial confinement that effectively prevented the migration and aggregation of Fe atoms, thereby securing their atomic dispersion. Simultaneously, the introduced Cu species participated in the formation of Cu-N bridges, which collaboratively modulated the local electronic structure and 3d electron state of the adjacent Fe-N_4_ centers. This dual strategy—where the cavity confinement ensured structural stability and atomic utilization, while the Cu-N doping optimized the electronic environment of the active sites—resulted in a Fe-Cu dual-atom catalyst with exceptional ORR activity and durability. The catalyst demonstrated outstanding performance in both flexible zinc-air batteries and proton exchange membrane fuel cells, showcasing how the deliberate integration of cavity confinement and heteroatom doping can create a synergistic enhancement far exceeding the sum of their individual effects. Similarly, Pan et al. [63] further exemplified this principle by designing a hollow porous dodecahedron nanocage with coordinated Fe, Cu, and Zn doping, where the combined cavity confinement and multi-metal synergy markedly enhanced the oxygen reduction activity. Figure 4a illustrates the synthesis process of this hollow nanocage structure. The subsequent subfigures (Figure 4b–g) provide a comprehensive characterization which confirm the uniform distribution of Fe, Cu, and Zn dopants throughout the hollow framework, demonstrating the successful fabrication of a multi-metal coordinated hollow structure.

#### 2.2.2. Internal-External Surface Heterojunctions

**Figure 5:**
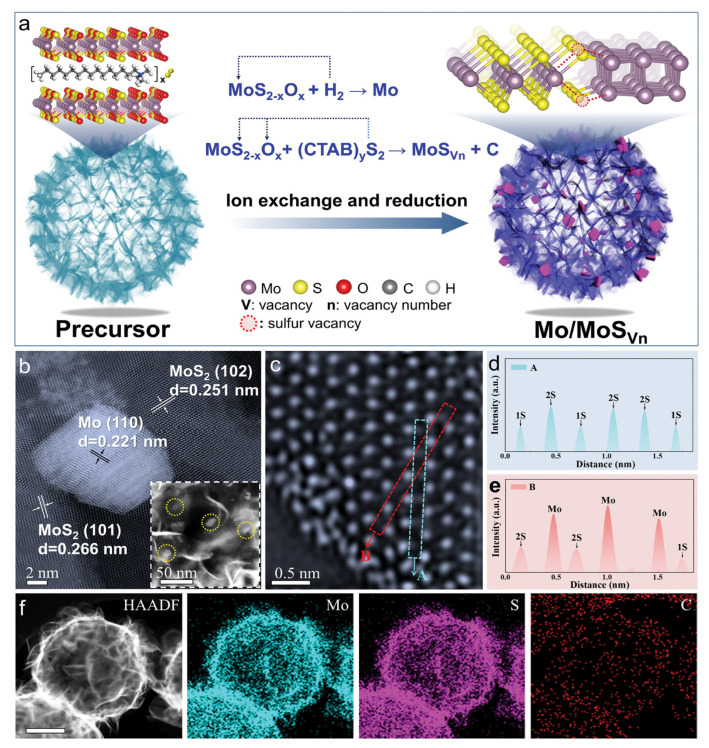
Synthesis and structural characterization of the hollow Mo/MoS_Vn_ nanoreactors. (**a**) Schematic illustration of the preparation process. (**b**–**f**) Morphological and compositional analyses, including AC-TEM, atomic intensity profiles, and HAADF-STEM with EDS mapping [64]. The scale bar in (**f**) is 100 nm.

Asymmetric functionalization of the internal and external surfaces of hollow structures to construct internal-external surface heterojunctions is another effective strategy for regulating the cavity microenvironment. The core advantage of this asymmetric design is the formation of a built-in electric field. The built-in electric field can regulate catalytic performance through three pathways: first, promoting directional charge transfer (from the inner surface to the outer surface or vice versa), optimizing the redox properties of active sites to adapt to the electronic needs of different reaction steps; second, forming a potential gradient to accelerate the migration of ions such as H^+^ and OH^−^ across the shell, reducing concentration polarization; third, realizing the spatial separation of redox reactions, effectively avoiding cross-reactions and side reactions of intermediate products [64,65]. A similar built-in electric field regulation strategy has been verified in hollow Mo/MoS_Vn_ nanoreactors [64]. The synthesis process (Figure 5a) illustrates the preparation of these hollow nanoreactors, while subsequent characterization (Figure 5b–f) confirms the presence of sulfur vacancies (SVs) within the MoS_2_ shell. By adjusting the SV concentration, the built-in electric field (BIEF) intensity can be tuned from 0.79 to 0.42 mV nm^−1^, exhibiting a parabolic relationship with both HER and urea oxidation reaction (UOR) activities. HER primarily occurs on the metallic Mo nanoparticles, while UOR mainly proceeds on the MoS_2_ shell, where SVs serve as the key active sites facilitating N–H bond cleavage. SV adjustment primarily benefits UOR by directly modifying the MoS_2_ shell electronic structure, but also indirectly influences HER through BIEF-mediated charge coupling across the heterojunction.

#### 2.2.3. Inner Surface Modification for Hydrophilic/Hydrophobic Microenvironments

For electrocatalytic reactions involving gas reactants or products such as ORR, OER, and HER, the hydrophilicity/hydrophobicity of the inner surface of the cavity directly affects proton/ion accumulation, gas bubble desorption, and reactant accessibility, and is a key surface property regulating catalytic performance [66,67]. Regulating the hydrophilicity/hydrophobicity of the inner surface through surface functionalization modification can construct a microenvironment suitable for specific reaction needs, realizing precise optimization of catalytic performance.

**Figure 6:**
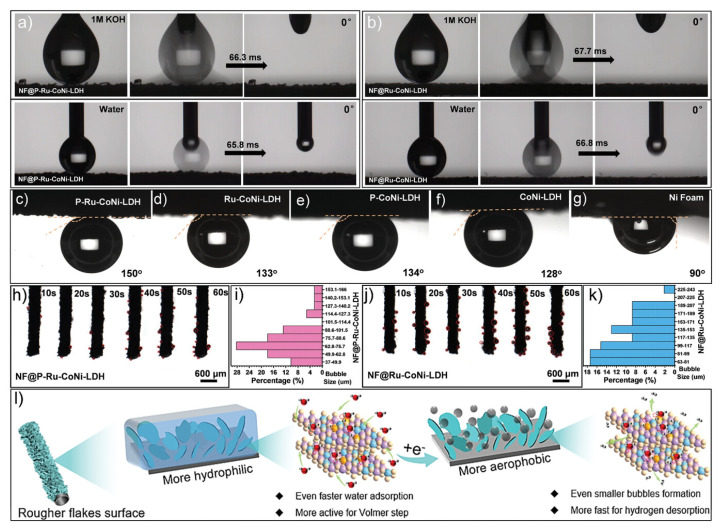
Wettability characterization, bubble behavior analysis, and proposed mechanism for the samples. (**a**,**b**) Contact angles of P-Ru-CoNi-LDH and Ru-CoNi-LDH were measured in 1 M KOH and water, respectively. (**c**–**g**) Hydrogen contact angles on various samples. (**h**,**i**) Sequential screenshots and corresponding bubble size statistics during the H_2_ evolution process on P-Ru-CoNi-LDH within 1 min. (**j**,**k**) Parallel screenshots and bubble size analysis for Ru-CoNi-LDH. (**l**) Schematic illustration of the mechanism for enhanced HER performance on P-Ru-CoNi-LDH [70].

Hydrophilic inner surfaces are usually achieved by introducing polar groups (e.g., -OH, -COOH, -SO_3_H) [68,69] or via specific surface treatments [70]. A representative example of such an engineering approach is the creation of superhydrophilic surfaces on hollow Ru-doped CoNi layered double hydroxide (LDH) nanotube arrays through air plasma treatment [70]. This surface engineering endows the catalyst with superhydrophilicity for rapid electrolyte infiltration and water adsorption (Figure 6a,b), as well as superaerophobicity to suppress the adhesion of in situ generated H_2_ bubbles (Figure 6c–g). The superhydrophilic surface strengthens the interaction between the catalyst and electrolyte, promotes proton/ion accumulation, and reduces concentration polarization. Meanwhile, the superaerophobic feature enables fast detachment and spillover of H_2_ bubbles with smaller sizes (Figure 6h–k), minimizing the blockage of active sites and lowering the kinetic barrier for the Volmer–Heyrovsky steps of HER. Notably, plasma treatment synergizes with electronic modulation induced by Ru doping, further accelerating water adsorption, charge transfer, and H_2_ spillover. Consequently, the optimized catalyst exhibits remarkably enhanced HER kinetics with an ultralow overpotential of 22 mV at 10 mA cm^−2^ in alkaline media, making it highly favorable for HER that demands fast proton supply and efficient gas release.

**Figure 7:**
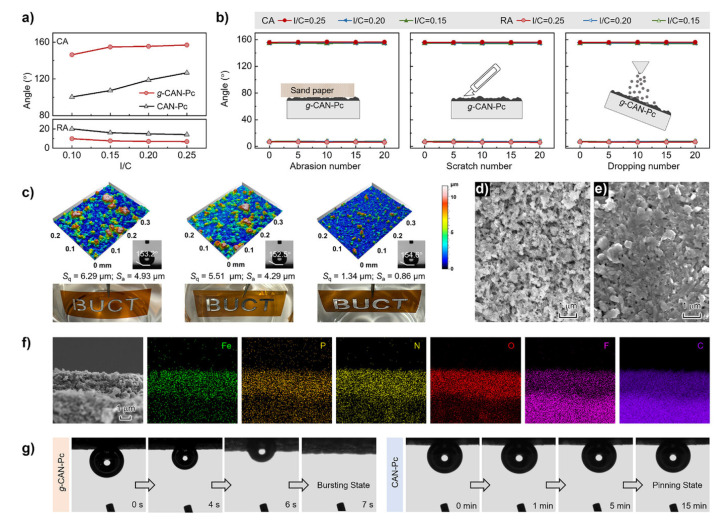
Comparative study of surface properties and processability for g-CAN-Pc and CAN-Pc derived catalyst layers. (**a**) Contact angle (CA) and rolling angle (RA) measurements under different ionomer/carbon (I/C) ratios. (**b**) Abrasion resistance tests against sandpaper, scalpel, and bead impact. (**c**) Evaluation of patterning fidelity via different printing methods. (**d**,**e**) SEM images of g-CAN-Pc and CAN-Pc layer surfaces. (**f**) Cross-sectional SEM view and elemental distribution of the g-CAN-Pc layer. (**g**) Snapshots of a single O_2_ bubble interacting with g-CAN-Pc and CAN-Pc electrodes in O_2_-saturated solution [73].

Hydrophobic inner surfaces are achieved by introducing non-polar groups (e.g., alkyl chains, fluorinated groups) [71,72]. Such groups can reduce the interaction energy between the inner surface and the electrolyte, promote the desorption of gas products (e.g., O_2_, H_2_), avoid active site blockage by bubbles, thereby improving the mass transport efficiency and long-term stability of OER and ORR. Di et al. confirmed this principle by their work on microenvironmental regulation of Fe─N_4_ sites [73]. In that study, fluorinated cyclotriphosphazene molecules were chemically grafted around the atomic Fe─N_4_ centers to create a fluorine-rich, hydrophobic nano-environment (Figure 7a). The material exhibits excellent mechanical stability against abrasion (Figure 7b). Different preparation methods yield various surface roughnesses but maintain consistent superhydrophobicity (Figure 7c). SEM and elemental mappings verify a uniform porous structure favorable for mass transport (Figure 7d–f). Importantly, O_2_ bubbles can rapidly penetrate the g-CAN-Pc layer within 7 s, while being trapped on conventional catalysts for 15 min (Figure 7g). This engineered hydrophobic shell effectively minimizes electrolyte flooding and critically facilitates the continuous supply of O_2_ gas to the active sites while promoting the removal of reaction products. This targeted regulation of the local environment directly addressed mass transport limitations, leading to a significant enhancement in both ORR activity and long-term stability in practical electrochemical devices, powerfully validating the utility of hydrophobic interfaces in optimizing gas-involving electrocatalysis.

Based on the advantages of hydrophilic and hydrophobic modifications, constructing amphiphilic gradient surfaces (hydrophilicity gradually decreases from the inner surface to the outer surface) has become a new design direction for bifunctional or trifunctional catalysts. Such gradient structures can simultaneously meet the needs of gas product desorption (hydrophobic outer layer) and ion transport (hydrophilic inner layer), providing an optimized microenvironment for multi-reaction synergistic catalysis such as ORR/OER [74].

### 2.3. Strain Effects and Dynamic Microenvironment

Traditional regulation of the cavity microenvironment mostly focuses on static structure and chemical properties, but in actual electrocatalytic reaction processes, hollow structures will undergo dynamic structural evolution under reaction conditions such as applied voltage and electrolyte infiltration, forming a dynamic microenvironment [75]. At the same time, the lattice strain induced by the inherent curvature of hollow structures also affects catalytic performance by adjusting the electronic structure of active sites [76]. Deeply understanding the evolution law of the dynamic microenvironment and the mechanism of strain effects is the key to realizing precise regulation of catalyst performance and long-term stability, and is also a frontier direction of current research.

#### 2.3.1. Shell Curvature-Induced Lattice Strain

**Figure 8:**
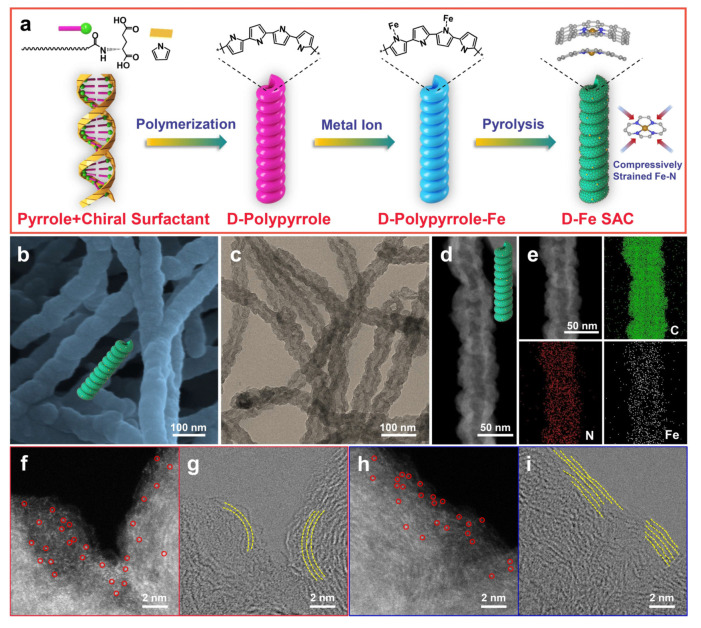
Synthesis and multi-scale characterization of the defect-rich Fe single-atom catalyst (D-Fe SAC). (**a**) Schematic illustration of the fabrication process. (**b**–**e**) Morphology and composition analysis: SEM image, TEM image, HAADF-STEM image, and corresponding elemental maps. (**f**–**i**) Aberration-corrected HAADF-STEM images at atomic resolution for D-Fe SAC (**f**,**g**) and its counterpart, LD-Fe SAC (**h**,**i**) [77].

The inherent curvature of hollow structure shells can generate tensile or compressive lattice strain, making strain engineering a pivotal strategy for regulating catalytic activity. This regulation is primarily achieved by modulating the d-band center of transition metals, thereby altering the adsorption energy of active sites and reaction intermediates. A compelling demonstration of this mechanism is found in the study on compressive strain modulation of single iron sites on a helical carbon support [77]. The helical carbon support is prepared via chiral surfactant self-assembly and pyrrole polymerization, retaining a right-handed helical morphology after pyrolysis (Figure 8a–c). Elemental mapping and aberration-corrected HAADF-STEM images further verify that Fe exists as isolated single sites (Figure 8e,f). Notably, the helical morphology induces edge curving of hollow nanofibers, forming abundant high-curvature carbon lattices (Figure 8g–i), where most single Fe sites are anchored. The high curvature of the hollow helical carbon support imposed significant compressive strain on the anchored Fe-N_4_ sites, resulting in a measurable contraction of the Fe–N bonds by approximately 1.5%. This geometric distortion directly induced a downward shift in the d-band center of the iron atoms. Consequently, the overly strong adsorption of key oxygen-containing intermediates (e.g., O*, OOH*) was effectively weakened, which not only significantly accelerated the rate-determining steps of the oxygen reduction reaction pathway but also suppresses active-site poisoning and structural degradation induced by overly strong intermediate binding. Such strain-induced optimization of intermediate adsorption strength simultaneously boosts ORR kinetics and enhances long-term operational stability, providing direct experimental validation for the deliberate use of curvature-induced strain to optimize electrocatalytic performance.

Conversely, tensile strain shifts the d-band center upward, adjusting the adsorption strength of H*, which is more suitable for HERs [78]. Du et al. [79] employed a rapid thermal quenching technique, which successfully introduced a substantial tensile strain of approximately 2.65% into the catalyst. This built-in strain played a pivotal role in modulating the local electronic structure, thereby optimizing the adsorption free energy of hydrogen intermediates (H*). Concurrently, the unique heterointerface between the amorphous and crystalline phases synergistically promoted water dissociation kinetics. The combined effects resulted in excellent HER performance in alkaline media, with activity rivaling that of precious-metal benchmarks. In addition, curvature-induced strain can also promote the formation of lattice defects, which themselves can serve as active sites, further improving catalytic activity.

#### 2.3.2. Operando Structural Evolution

Under actual electrocatalytic reaction conditions, hollow nanostructures will undergo dynamic structural evolution, commonly referred to as electrochemical reconstruction. This evolution will change the properties of the cavity microenvironment, thereby affecting catalytic performance and stability. Electrochemical reconstruction is a complex dynamic process, mainly including three core processes: surface reconstruction, cavity volume change, and defect migration and repair [80,81]. Surface reconstruction is the most common phenomenon in transition metal-based hollow catalysts. For example, during the OER, the surface of Ni, Co-based hollow structures will be oxidized to form hydroxide phases such as NiOOH and CoOOH. Such hydroxide phases have been confirmed to be the real active species for OERs [82]. Cavity volume change is an “electrochemical breathing” phenomenon, that is, the cavity volume expands and contracts periodically with the progress of redox reactions. This volume change can adjust the shell strain state and mass transport path, indirectly affecting catalytic performance [83]. Defect migration and repair is a self-optimization processes of the catalyst. During the reaction, defects will migrate to positions with higher activity (these high-activity positions are inherently defective sites or can form stable defective structures after defect migration) or achieve defect repair through atomic rearrangement, thereby balancing catalytic activity and structural stability [84].

**Figure 9:**
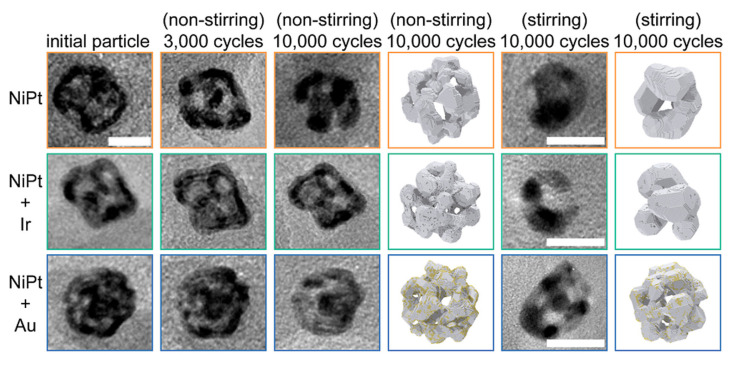
Comparative study of electrochemical coarsening in np-NiPt alloys under different electrolyte conditions. Identical-location TEM (IL-TEM) imaging and kinetic Monte Carlo (kMC) simulations reveal the coarsening mechanisms of np-NiPt, np-NiPt+Ir, and np-NiPt+Au during accelerated stress tests (0.6–1.1 V vs. RHE). The left panel shows results in a static electrolyte, while the right panel demonstrates the effect of a stirred electrolyte (introducing Pt^2+^ concentration gradients) after 10,000 cycles. Scale bar: 10 nm [86].

Although the importance of electrochemical reconstruction has been recognized, current research still faces key characterization challenges: how to accurately distinguish pre-designed static active sites from dynamically reconstructed active sites, and how to real-time track the dynamic evolution of the cavity microenvironment under reaction conditions. In situ/operando characterization technology is the core means to solve this challenge. Among them, in situ X-ray absorption spectroscopy can be used to track the valence state changes and coordination environment evolution of metal active sites, and in situ transmission electron microscopy can intuitively observe the dynamic changes in cavity structures. For example, Xiao et al. [85] used in situ liquid-phase TEM technology to successfully reveal the structural transformation mechanism of MOF-derived hollow carbon-based catalysts during CO_2_ electrochemical reduction, confirming the dynamic migration and redispersion of metal nanoparticles during the reaction, and the regulatory effect of diffusion rate on product morphology. In another notable application of advanced operando imaging, Li et al. [86] employed identical-location liquid-phase TEM (IL-LPTEM) combined with kinetic Monte Carlo simulations to atomically resolve the origin of morphological instability in topologically complex nanoporous alloys (Figure 9). Their work specifically unveiled two co-existing electrochemical coarsening pathways—potential-driven dissolution/redeposition and surface diffusion of low-coordination atoms—and demonstrated that trace element doping could selectively suppress these degradation mechanisms.

However, existing in situ characterization technologies still have limitations, including insufficient time resolution that makes capturing rapid reconstruction processes difficult, inadequate spatial resolution that hinders the observation of atomic-level dynamic changes, and poor electrolyte compatibility that prevents some technologies from working in real electrolyte environments. These limitations impede the comprehensive understanding of the dynamic evolution of the cavity microenvironment. In the future, the development of in situ characterization technologies with high spatiotemporal resolution and high sensitivity, combined with theoretical calculations such as first-principles and molecular dynamics simulations, will be the key direction to reveal the mechanism of dynamic microenvironment evolution and realize the precise design of catalysts.

## 3. Controllable Synthesis Strategies for Targeted Cavity Microenvironments

The rational design of cavity microenvironments depends on advanced controllable synthesis strategies, which allow precise tuning of the geometry, composition, and interface of hollow nanostructures to meet specific electrocatalytic requirements. Various synthetic approaches have been developed for hollow structures, each with distinct advantages and limitations. Template-mediated synthesis offers good control over structure and morphology but involves complex procedures and scalability issues. Template-free methods, including Ostwald ripening, Kirkendall effect, galvanic replacement, selective etching, and dynamic self-assembly, simplify the fabrication process, yet they often suffer from limited uniformity, strict reaction conditions, narrow material scope, or insufficient reproducibility [27,87,88]. Therefore, the selection of a synthetic strategy must match the microenvironmental demands of target electrocatalytic reactions. The ultimate goal of controllable synthesis is to establish precise correlations among synthesis parameters, cavity microenvironment, and catalytic performance.

### 3.1. Synthesis-Parameter-Microenvironment-Performance Correlation

Mechanical stability is a prerequisite for the long-term service of hollow electrocatalysts, especially under harsh reaction conditions, including high potential, strong acid/alkali electrolytes, and gas bubble scouring. The template-mediated synthesis coupled with high-temperature annealing represents an effective strategy for enhancing mechanical stability: annealing can improve the shell crystallinity and reduce internal defects, thus boosting structural rigidity. This mechanistic advantage has been concretely validated in a seminal study by Mezzavilla et al. [89], who systematically investigated Pt_x_Ni_y_ nanoparticles confined within hollow graphitic spheres for ORR. They found that high-temperature annealing at ≥850 °C was pivotal for fabricating a complete and robust graphitic shell; this thermally engineered encapsulation not only optimized catalytic activity but also endowed the nanoparticles with exceptional resistance to sintering and degradation during durability tests.

**Figure 10:**
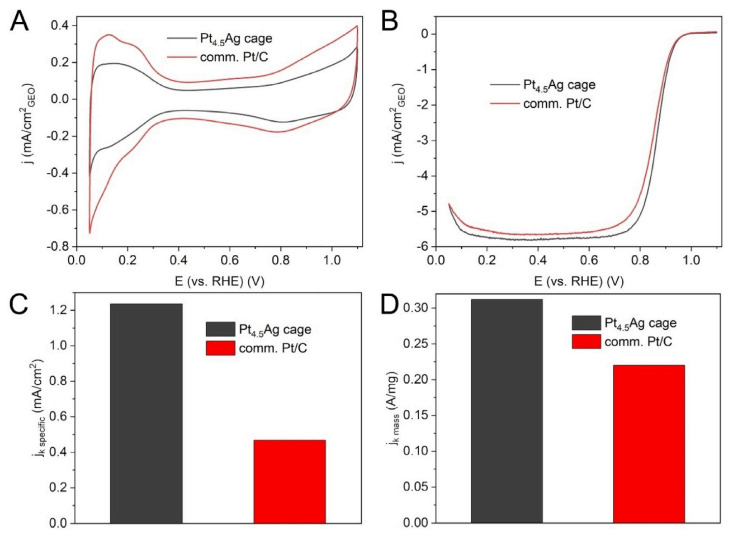
(**A**,**B**) Cyclic voltammograms and ORR polarization curves of the carbon-supported Pt_4.5_Ag icosahedral nanocages versus commercial Pt/C. (**C**,**D**) Corresponding specific and mass activities presented as kinetic current densities (jₖ), normalized to the electrochemical surface area (ECSA) and Pt mass, respectively. All currents in (**A**,**B**) are normalized to the geometric electrode area (0.196 cm^2^) [90].

Mass transport efficiency is another core factor governing catalytic performance, which is tightly associated with the porous structure and cavity geometry of the hollow shell. Galvanic replacement and selective etching stand out as efficient methods for constructing high-porosity shell structures with interconnected transport channels, which can minimize the diffusion resistance of reactants and products and maximize the utilization of active sites. For the ORR, Wang et al. [90] reported a typical case that exemplifies this merit, involving the synthesis of hollow Pt-Ag icosahedral nanocages. In their work, the nanocages were fabricated through the epitaxial growth of a Pt layer on Ag icosahedral seeds, followed by selective etching of the Ag core. This process created a well-defined porous and hollow architecture, which contributed to a mass-transfer-friendly environment. Electrochemically, the catalyst was first activated in Ar-saturated 0.1 M HClO_4_ solution, with typical hydrogen adsorption peaks indicating accessible active surface sites (Figure 10A). ORR polarization curves in O_2_-saturated 0.1 M HClO_4_ solution (Figure 10B) and subsequent kinetic calculations (Figure 10C,D) showed that these structurally engineered nanocages demonstrated a specific activity nearly three times higher than that of conventional Pt/C catalysts. The enhanced performance underscores how the tailored porosity and cavity geometry achieved via etching-based synthesis can effectively facilitate reactant/product diffusion and increase the accessibility of active sites, thereby directly translating structural advantages into catalytic superiority.

The electronic structure of active sites directly affects the adsorption and desorption energy of reaction intermediates, and its regulation can be effectively achieved through MOF-derived synthesis and doping modification. MOF-derived Fe-N-C hollow nanostructures are classic examples of electronic structure regulation, and molecular-level precursor engineering provides a powerful means to precisely control this process. Recent work on Fe- and N-co-doped carbon nanotubes derived from a rationally designed bimetallic complex offers a notable illustration of this strategy [91]. This study employed a multi-ligand coordinated self-assembly strategy to create an ordered precursor (FeZn-PBMI), which upon pyrolysis successfully yielded gram-scale quantities of a catalyst comprising FeN_x_ sites and Fe_x_C atomic clusters decorated on N-doped carbon nanotubes (Fe_x_C@FeNCNTs). The ordered distribution of Zn and Fe within the precursor effectively prevented Fe aggregation during high-temperature pyrolysis. As a result, the Fe_x_C@FeNCNTs catalyst exhibited a superior ORR half-wave potential of 0.87 V (vs. RHE), surpassing that of commercial Pt/C (0.85 V), and demonstrated excellent long-term stability over 1000 cycles in a Zn–air battery.

### 3.2. Emerging Advanced Synthesis Techniques

With the in-depth demand for precise regulation of cavity microenvironments, a series of emerging advanced synthesis techniques have been developed in recent years. These techniques break through the limitations of traditional methods in terms of structural controllability and functional customization, providing new approaches for the construction of high-performance hollow electrocatalysts. The following section focuses on three representative emerging techniques: liquid droplet template synthesis, electrochemical deposition with gas bubble templates, and machine learning-guided synthesis, and elaborates on their synthesis mechanisms, advantages, and application progress.

**Figure 11:**
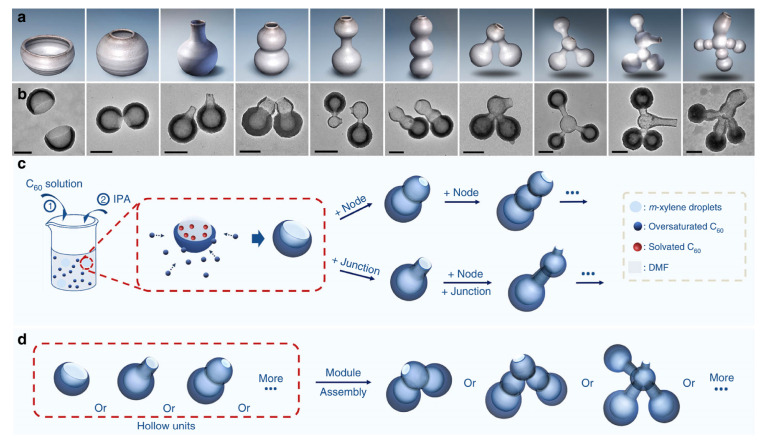
Schematic illustrations of the design and synthesis. (**a**,**b**) Corresponding pottery models and TEM images of the created nanostructures via nanopottery. (**c**,**d**) Schematics showing the synthesis and subsequent connection of individual hollow units, respectively. Scale bars: 200 nm [93].

Liquid droplet template synthesis, as a novel soft template method, utilizes liquid droplets such as water-in-oil emulsions and microfluidic droplets as sacrificial templates to construct hollow structures with adjustable inner surfaces [92]. The core advantage of this technique lies in the molecular-level controllability of the template, which can effectively adjust the cavity morphology by regulating the size and shape of the droplets. This exceptional programmable capability is vividly exemplified in a landmark study by Chen et al., [93] which proposed a “nanoceramics” synthesis strategy using xylene droplets as dynamic liquid templates and fullerene (C_60_) molecules as shell precursors. The size of the droplets is typically controlled by varying emulsification parameters such as shear force and phase ratios, while the shape is dynamically modulated through interfacial tension manipulation and mechanical deformation prior to shell solidification. As shown in Figure 11a–d, through the interfacial self-assembly and cross-linking of C_60_ at the oil-water interface, they achieved the on-demand fabrication of hollow nanostructures with unprecedented morphological complexity, including bowls, bottles, gourds, and even interconnected multi-compartment systems. This work exemplifies how the precise manipulation of droplet behavior translates directly into tailored cavity geometry, pushing the frontier of soft-templating from simple spheres to architecturally sophisticated and functional hollow materials.

**Figure 12:**
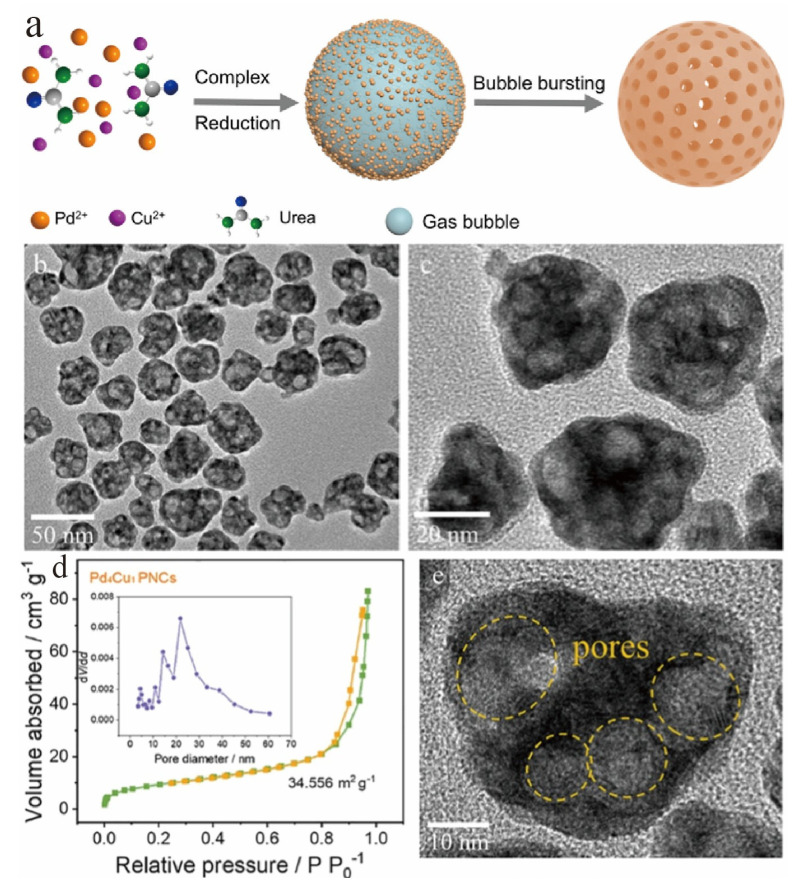
Synthesis and structural characterization of Pd_4_Cu_1_ nanocages. (**a**) Schematic illustration of the formation mechanism; (**b**,**c**) TEM images at different magnifications; (**d**) N_2_ adsorption–desorption isotherms and the corresponding pore-size distribution curve (inset); (**e**) HRTEM image [95].

Electrochemical or chemical deposition with gas bubble templates is a green and efficient synthesis method that directly grows hollow structures on the surface of electrodes using in situ generated gas bubbles as templates [94]. This technique can realize the synchronous construction of hollow structures and electrode integration, and the obtained hollow structures have open ends, which is conducive to gas diffusion and bubble detachment. Beyond electrode-bonded structures, the bubble-template strategy also exhibits remarkable versatility for powder catalyst synthesis, and this broad applicability is strikingly demonstrated in the fabrication of Pd_4_Cu_1_ porous nanocages for formic acid electrooxidation [95]. In this work, the thermal decomposition of urea served as an in situ source of CO_2_ and NH_3_ gas bubbles (Figure 12a), which acted as dynamic soft templates to guide the assembly of alloy nanoparticles into three-dimensional nanocages (Figure 12b,c) with a hollow interior and ultrathin walls (~2.1 nm). This novel architecture provided a large active surface area (Figure 12d,e) and efficient mass transport pathways, resulting in a mass activity 4.8 times higher than that of commercial Pd/C and significantly improved durability. This case underscores that the gas-template approach, whether in electrochemical or chemical deposition, is a powerful and generalizable paradigm for creating structurally advantageous hollow nanomaterials that address key challenges in electrocatalysis, such as active site accessibility and reactant/product diffusion.

**Figure 13:**
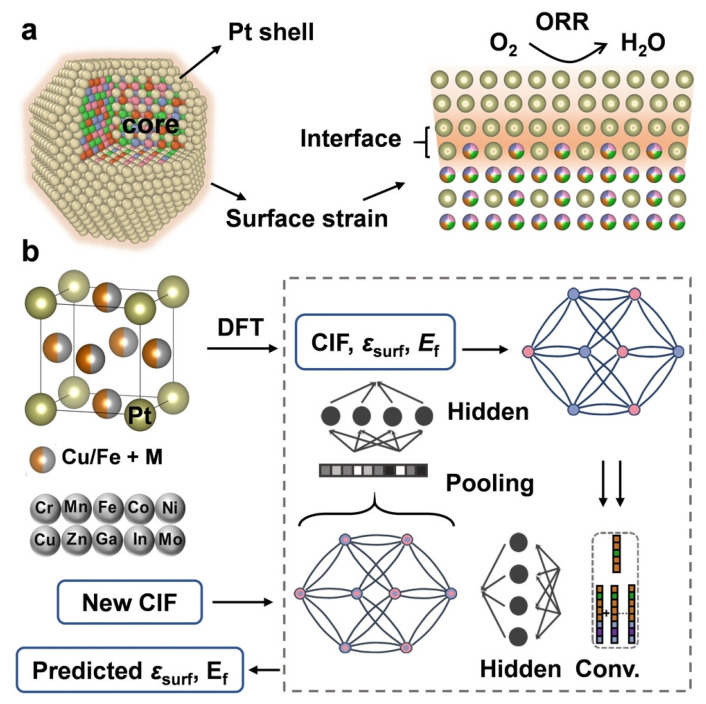
(**a**) Origin of surface strain (ε_surf_) from lattice mismatch between the intermetallic/alloy core and Pt shell. (**b**) Machine learning-guided element selection for low-Pt ORR catalysts, with the gray box representing the CGCNN model [98].

Machine learning (ML)-guided synthesis has become a revolutionary tool in the field of catalyst design, which can efficiently predict the optimal synthesis parameters by establishing the quantitative relationship between synthesis parameters, microenvironmental properties, and catalytic performance, thereby avoiding the traditional time-consuming trial-and-error process [96]. A universal meta-learning framework was developed by Chen et al. to accelerate the identification of HER catalysts spanning diverse alloy families [97]. By leveraging knowledge from large theoretical datasets and transferring it to data-scarce systems, this approach successfully identified non-precious alloy compositions, one of which was experimentally validated to exhibit excellent HER activity, completing a full prediction-synthesis loop. Furthermore, ML’s unparalleled ability to navigate high-dimensional compositional spaces is further illustrated in the discovery of low-platinum high-entropy intermetallic compounds (HEIs), specifically Pt(FeCoNiCu)_3_, for ORR [98]. For Pt-based intermetallic catalysts, surface strain (*ε*_surf_)—derived from lattice mismatch between the intermetallic core and Pt shell and determinable from lattice parameters (Figure 13a)—dominates ORR kinetics by modulating oxygen-containing intermediate adsorption and dissociation, while formation energy (*E*_f_) correlates with catalyst stability. Thus, *ε*_surf_ and *E*_f_ were selected as target values for the ML process. Figure 13b illustrates the DFT-based and ML-accelerated element selection process: PtM_3_-type intermetallics were designed with Fe, Cu, Mn as main non-Pt elements, various 3d transition metals and Ga, In, Mo as substitutes (at different ratios), and six random structures per ratio were used to construct the ML dataset, with DFT calculations providing *ε*_surf_ and *E*_f_. Herein, a crystal graph convolutional neural network was employed to expedite the multicomponent design based on a first-principles dataset, achieving high prediction accuracy for key properties. The carbon-supported Pt(FeCoNiCu)_3_ catalyst, synthesized via a freeze-drying-annealing method, demonstrated exceptional performance with an ultrahigh mass activity of 4.09 A mg_Pt_^−1^ and a specific activity of 7.92 mA cm^−2^, alongside significantly enhanced electrochemical stability attributed to the sluggish diffusion effect within the HEIC structure. Although the examples above focus on using machine learning to optimize metal compositions, they underscore the broader potential of data-driven methods to establish synthesis–parameter–microenvironment–performance correlations. Current machine learning models are mostly limited to screening dopant species, doping ratios, and simple synthetic parameters, while accurate prediction of complex structural features (e.g., precise cavity size, shell curvature, surface defects, and multi-component synergy) remains challenging due to insufficient high-quality labeled data and unclear structure–activity mechanisms. With the growth of standardized synthetic data and advanced AI model architectures, machine learning is poised to directly guide the rational design of synthesis protocols—for instance, by predicting optimal reaction conditions for achieving desired cavity geometries and surface functionalities—thereby accelerating the development of advanced hollow electrocatalysts.

## 4. Application in Electrocatalysis: Microenvironment-Performance Correlations

Hollow nanomaterials, featuring unique cavity-confined microenvironments, have emerged as promising electrocatalyst platforms due to their tunable structural parameters, enhanced mass transport efficiency, and regulated surface electronic properties. Rational modulation of the cavity microenvironment—encompassing structural geometry, surface wettability, chemical composition, and interfacial interactions—enables precise tailoring to the intrinsic requirements of diverse electrocatalytic reactions.

### 4.1. Oxygen Reduction Reaction

ORR serves as the core cathodic reaction in fuel cells and metal–air batteries, whose catalytic efficiency is predominantly limited by sluggish reaction kinetics, inefficient O_2_ mass transport, and unsatisfactory stability of active sites. The multi-electron transfer process of ORR involves the adsorption and desorption of oxygen-containing intermediates (OOH*, O*, OH*), and the cavity microenvironment of hollow nanomaterials can precisely regulate these elementary steps through synergistic modulation of mass transport, electronic structure, and active site confinement [59].

The design of cavity microenvironments for ORR adheres to the principle of demand-oriented precise matching, in which structural and chemical parameters are tailored to resolve the core bottlenecks of the electrocatalytic process [99,100]. To fulfill the microenvironmental requirement for fast O_2_ diffusion, a rational design featuring a hierarchical pore network and a large cavity-to-shell volume ratio can be adopted, which effectively promotes mass transport and thereby delivers a high limiting current density. To construct an electron-rich surface microenvironment, the engineering of N/P-doped carbon shells and metal-N_x_ active sites favors the achievement of a positive onset potential. For the stabilization of active sites, the integration of confined single-atom catalysts and a conformal carbon coating strategy significantly improves catalyst durability, suppressing performance decay over long-term cycling. With regard to the microenvironmental demand for efficient proton accessibility, rational modifications—including a hydrophilic inner surface and gradient porosity—can be introduced to promote high selectivity toward the 2-electron or 4-electron ORR pathway.

**Figure 14:**
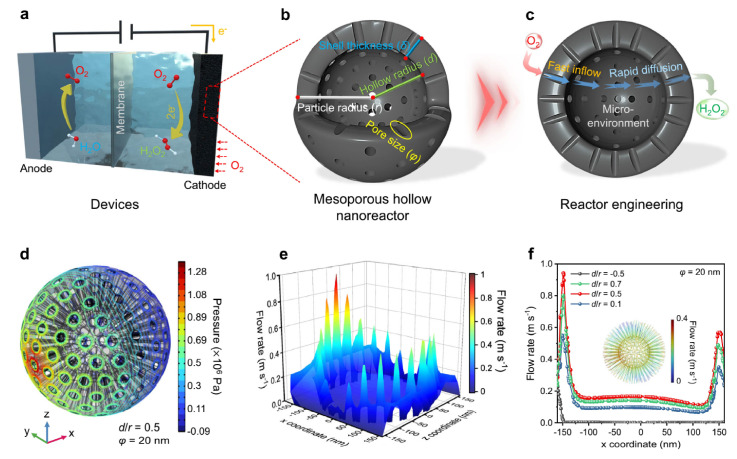
Design and simulated fluid dynamics of hollow mesoporous carbon nanoreactors for the 2e^−^ ORR to H_2_O_2_. (**a**–**c**) Schematic of the nanoreactor concept. (**d**) Pressure contour distribution within the optimal structure (d/r = 0.5, φ = 20 nm). (**e**) 3D cross-sectional flow velocity mapping. (**f**) Flow velocity as a function of hollow ratio (d/r), showing maximized transport at d/r = 0.5 (inset: flow profile) [101].

For efficient 4-electron ORR, Zhao et al. [100] demonstrated that rational design of the hollow cavity microenvironment of PtCo alloy nanorods enables strong confinement effects to stabilize active sites, optimize the adsorption–desorption of key ORR intermediates, and suppress the generation of H_2_O_2_ byproducts. By precisely matching the structural and electronic demands of complete oxygen reduction, the cavity-confined electrocatalysts exhibit outstanding 4-electron selectivity, high activity, and enhanced long-term durability, fully embodying the “demand-oriented precise matching” principle. By contrast, when targeting H_2_O_2_ production via the 2-electron ORR pathway, the microenvironment needs to be tailored to favor the formation and desorption of OOH intermediates. Tai et al. [101] provide a quintessential case study of the aforementioned “demand-oriented precise matching” principle for the 2-electron ORR pathway. They engineered mesoporous carbon spheres with programmable interiors (N-MCSs) as efficient nanoreactors for H_2_O_2_ electrosynthesis (Figure 14a–c). Fluid dynamics simulations showed that the mesoporous channels induce a pressure drop, driving electrolyte flow into the sphere interior to expand catalytic surface contact, and generate fluid acceleration to intensify diffusion, facilitating rapid O_2_ delivery and H_2_O_2_ exhaustion (Figure 14d–f). By systematically comparing solid, yolk-shell, and hollow structures, their work identified that the completely hollow architecture (H-N-MCSs) delivered optimal performance, achieving an H_2_O_2_ selectivity of 92.6% in 0.1 M KOH and an industrial-scale current density of 300 mA cm^−2^ in a flow cell. This superior efficacy originates from the confined microenvironment created by the hollow structure: the highly porous N-doped carbon shell ensures rapid mass transport, while the enclosed cavity acts as a nanoreactor that effectively concentrates and confines O_2_ reactants. This enhances the local O_2_ concentration and prolongs its contact with the active sites, thereby optimizing the adsorption/desorption energetics of the critical *OOH intermediate and steering the reaction efficiently toward the 2-electron pathway.

**Figure 15:**
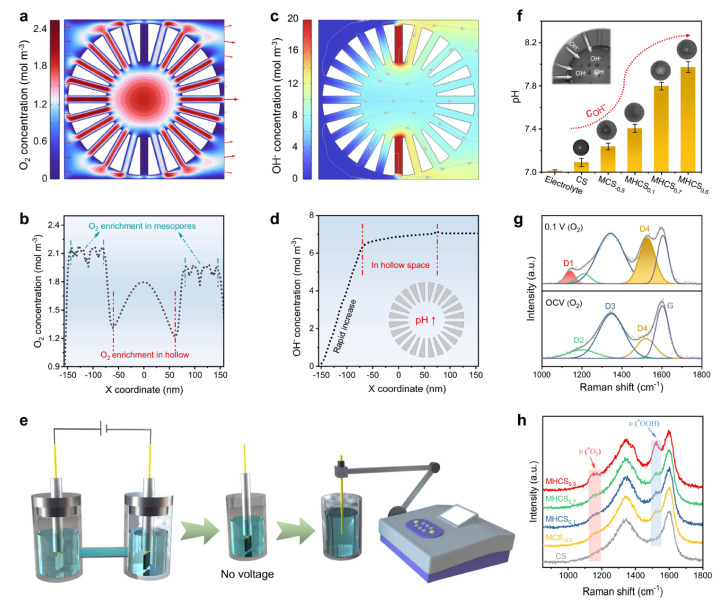
Concentration profiles and surface characterization of mesoporous carbon nanoreactors. (**a**–**d**) Simulated spatial and cross-sectional distributions of O_2_ (**a**,**b**) and OH^−^ (**c**,**d**) concentrations within the model (d/r = 0.5, φ = 20 nm). (**e**,**f**) Schematic and results of OH^−^ concentration tests on the electrode surface. (**g**,**h**) In situ Raman spectra of the electrodes at open circuit and under applied potential (0.1 V vs. RHE). [101].

Furthermore, to elucidate the mechanistic origin of this performance and achieve precise design, the study employed finite element simulations to optimize the diffusion-related geometric parameters of the mesoporous hollow nanospheres (MHNs), including the hollow-to-radius ratio (d/r) and the pore size (φ). The simulations revealed a phenomenon of “localized diffusion intensification” within the mesoporous channels. As shown in Figure 15, an optimal structure with d/r = 0.5 and φ = 20 nm was identified, which maximized the fluid velocity through the porous shell. This specific geometry ensures the rapid influx of O_2_ and the efficient expulsion of the produced H_2_O_2_ from the active sites, preventing its accumulation and further reduction. The internal hollow space also functions as a fluid buffer zone, contributing to a stable internal microenvironment. Specifically, high fluid flow rates in the mesoporous channels promote O_2_ enrichment (Figure 15a,b), which facilitates O_2_ activation and *OOH intermediate formation. The generated OH^−^ by-products accumulate as a flow buffer, leading to a localized pseudo-alkaline microenvironment (Figure 15c,d), experimentally verified by pH measurements showing higher OH^−^ concentrations on the optimal MHCS_0.5_ electrode (Figure 15e,f). In situ Raman spectroscopy further confirmed that O_2_ enrichment and local pH elevation accelerate O_2_ activation, stabilize *O_2_ intermediates, and inhibit *OOH protonation (4e^−^ pathway), thereby promoting H_2_O_2_ generation, with the most pronounced ν_*O2_ and ν_*OOH_ signals observed for the optimal structure (Figure 15g,h).

### 4.2. Oxygen Evolution Reaction

Following the in-depth discussion on ORR, the regulatory role of the cavity microenvironment in another pivotal electrocatalytic reaction—OER—deserves equal attention. OER serves as the anodic reaction in water electrolysis and rechargeable metal–air batteries, characterized by a high overpotential requirement and harsh reaction conditions such as high potential and alkaline electrolyte, along with the generation of oxygen bubbles. The core challenges for OER catalysts include efficient bubble management, accelerated hydroxide ion (OH^−^) transport, and stabilization of high-valence metal active species [102]. The cavity microenvironment can address these challenges through precise modulation of structural morphology, surface wettability, and chemical composition, thereby enhancing OER performance.

**Figure 16:**
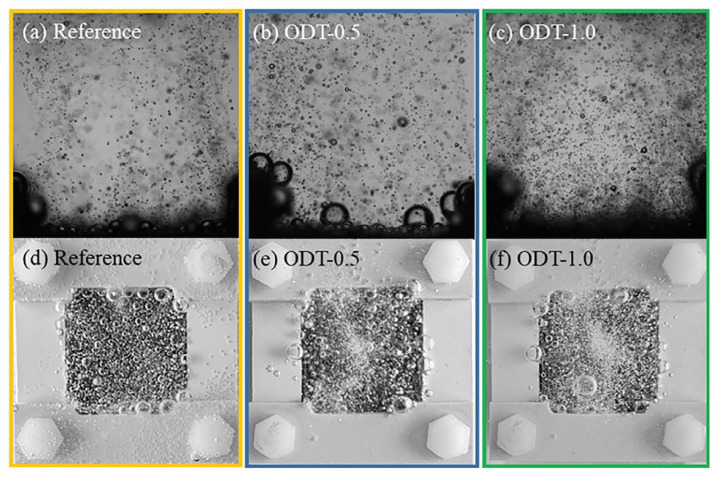
Bubble visualization for (**a**,**d**) the conventional ACL, (**b**,**e**) ODT-0.5, and (**c**,**f**) ODT-1.0 in the transparent three-electrode system with the utilization of a high-speed camera from the front side and a DSLR from the upper side [104].

Bubble management is a critical issue for OER catalysts, as bubble adhesion on the catalyst surface can block active sites and hinder reaction progress [103]. The cavity structure of hollow nanomaterials can serve as both nucleation sites and escape channels for oxygen bubbles, effectively reducing bubble blockage. Zhao et al. [104] provided a significant macro-scale structural strategy termed “Agglomerate Engineering” to address severe bubble blockage in proton exchange membrane water electrolyzers operating at high current densities. Their work focused on engineering the IrO_x_-ionomer agglomerates within the anode catalyst layer. By employing self-assembling nanotechnology, they successfully introduced interconnected submicron pores and nanocavities into the agglomerates, a structure confirmed by synchrotron radiation-based nano-CT, TEM, and BET analysis. This engineered porous architecture functioned as controlled nucleation sites and efficient escape channels, which enhanced both dissolved oxygen and bubble transport, as verified by RDE tests and in situ bubble visualization (Figure 16a–f). The agglomerate engineering yielded remarkable performance enhancements: the mass transport overpotential was drastically reduced from 330 mV to only 30 mV at 5 A cm^−2^. Furthermore, the PEMWE achieved high operating current densities of 5 A cm^−2^ at 2.04 V with a Nafion 115 membrane and 7 A cm^−2^ at 2.07 V with a Nafion 212 membrane, using a low catalyst loading of 0.72 mg_Ir_ cm^−2^.

The cavity structure also optimizes mass transport during OER. It can establish a concentration gradient of OH^−^, accelerating the migration of OH^−^ to active sites. Simultaneously, the bubble escape channels provided by the cavity prevent bubble aggregation, ensuring unobstructed mass transport. SnO_2_/NiO hollow nanotubes with a hierarchical pore structure were engineered as a representative system that exemplifies these dual mass transport advantages [105]. Synthesized via electrospinning and calcination, this three-dimensional hollow nanotube framework directly addresses bubble management by providing efficient pathways for the rapid release of oxygen bubbles, preventing site blockage and ensuring unobstructed electrolyte penetration. Concurrently, the material integrates n-p heterojunctions and enriched oxygen vacancies at the SnO_2_/NiO interface. This electronic structure modulation optimizes the adsorption of reaction intermediates and, as revealed by in situ Raman studies, dramatically lowers the energy barrier for the formation of active NiOOH species. This synergy between structural and electronic engineering resulted in exceptional OER performance: a low overpotential of 200 mV at 10 mA cm^−2^ and robust stability for over 90 h.

### 4.3. Hydrogen Evolution Reaction

HER is the core step for hydrogen production via water electrolysis, and its catalytic efficiency is restricted by three key factors: reducing reaction overpotential, accelerating proton transport, and promoting rapid detachment of generated hydrogen bubbles [106]. Similar to ORR and OER, the cavity microenvironment of hollow nanomaterials can effectively optimize these issues through rational regulation of surface wettability, electronic structure, and mass transport, thereby improving HER performance.

Notably, the regulation of surface wettability for HER requires two diametrically opposite design criteria. On the one hand, a hydrophilic surface is crucial for HER, especially in acidic electrolytes. It increases local proton concentration, reduces reaction resistance from concentration inhomogeneity, and accelerates the reaction rate. According to the Nernst equation, each order of magnitude increase in local proton concentration reduces the overpotential by approximately 59 mV. On the contrary, the detachment rate of hydrogen bubbles, another critical factor affecting HER performance, demands an aerophobic surface, which favors the rapid desorption of hydrogen bubbles to avoid catalyst active site blocking. To reconcile these contradictory requirements and simultaneously overcome the bottlenecks of insufficient proton supply and sluggish product desorption, a rational cavity microenvironment design—hydrophilic and aerophobic surface—can simultaneously address the issues of proton supply and product desorption, integrating the advantages of both wettability characteristics to achieve synergistic enhancement of HER catalytic performance [107].

Such a design principle has been effectively implemented and experimentally validated in a recent study on self-standing hollow Ni-doped Mo_2_C nanotube arrays (Ni-Mo_2_C HNAs) [108]. In this work, the researchers rationally engineered a cavity-rich microstructure by constructing vertically aligned hollow nanotubes through the Kirkendall diffusion process during annealing. The resulting three-dimensional hollow architecture inherently offers a large specific surface area and abundant internal cavity space, which not only facilitates efficient electrolyte penetration and sufficient proton supply (fulfilling the hydrophilic function) but also provides unobstructed channels for the rapid release of generated H_2_ bubbles (realizing the aerophobic function). Furthermore, Ni doping effectively modulates the electronic structure of Mo_2_C, weakening the adsorption strength of hydrogen intermediates and further accelerating the kinetics of product desorption. Consequently, this integrated material design achieves exceptional HER performance, exhibiting low overpotentials of 58 mV in acidic electrolyte and 92 mV in alkaline electrolyte at 10 mA cm^−2^. Moreover, the catalyst shows only a 34 mV increase in overpotential after 12 h of continuous operation in 1 M KOH, demonstrating outstanding long-term stability and fast reaction kinetics.

**Figure 17:**
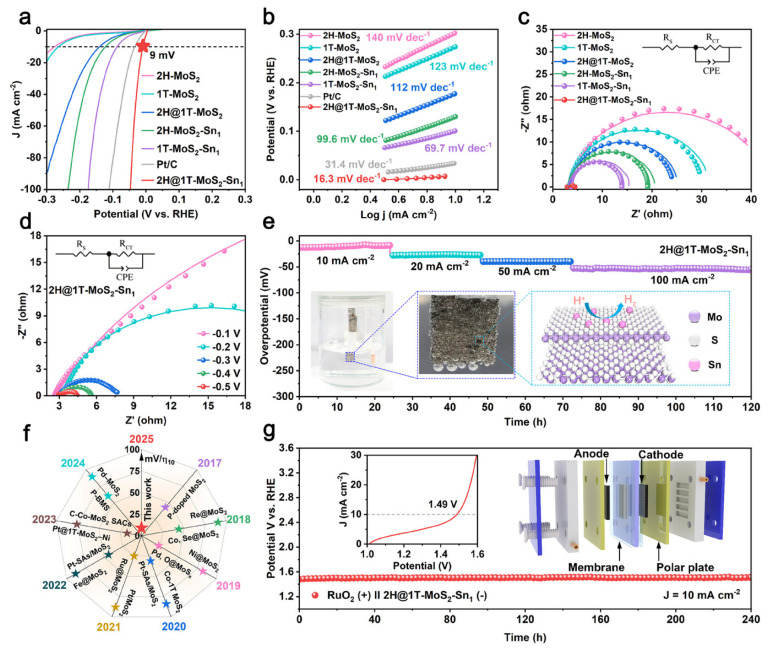
Electrochemical HER performance in acidic electrolyte. (**a**) Polarization curves. (**b**) Corresponding Tafel plots. (**c**) Nyquist plots. (**d**) Nyquist plots of the 2H@1T-MoS_2_-Sn_1_ nanoreactor at different potentials. (**e**) Chronoamperometric stability tests at various current densities. (**f**) Comparison of overpotentials with reported MoS_2_-based catalysts. (**g**) Performance of a PEM water electrolyzer using the 2H@1T-MoS_2_-Sn_1_ nanoreactor (cathode) and commercial RuO_2_ (anode) at 10 mA cm^−2^. Inset: Overall water-splitting polarization curve [109].

In addition to surface wettability regulation, the cavity microenvironment can optimize the electronic structure of the catalyst to enhance HER performance. The interfacial charge transfer between different components in hollow heterostructures modulates the adsorption free energy of hydrogen atoms (ΔG), a key descriptor for HER performance. The ideal ΔG is close to 0 eV, which balances the adsorption and desorption of H*. Such synergistic regulation underpins the high HER activity of hollow MoS_2_-M heterostructures, and this mechanism is further advanced in a study focusing on a hollow core–shell 2H@1T-MoS_2_-Sn_1_ nanoreactor tailored for acidic HER [109]. In this catalyst, Sn single atoms are anchored on the shell of a 2H@1T-MoS_2_ Mott–Schottky phase junction. The hollow core–shell architecture itself provides a rich microenvironment for mass transport and active-site exposure. The outstanding performance stems from a dual synergistic mechanism. The Mott–Schottky junction formed at the 2H/1T phase interface creates a strong built-in electric field that drives efficient and spontaneous electron transfer across the interface, optimizing charge transfer kinetics. More importantly, the Sn single atoms, through the Sn-S_2_-Mo coordination motif, markedly modulate the electronic structure of the Mo atoms, inducing a distinct shift in the d-band center and thereby precisely tuning the hydrogen adsorption/desorption process. This simultaneous engineering of interfacial charge transfer and surface catalytic sites enables the catalyst to achieve a breakthrough HER performance in acidic medium (Figure 17a–g), requiring an ultralow overpotential of only 9 mV to reach 10 mA cm^−2^ and exhibiting an extremely small Tafel slope of 16.3 mV dec^−1^—the best performance reported to date among MoS_2_-based electrocatalysts.

### 4.4. Multifunctional and Tandem Catalysis

Beyond single electrocatalytic reactions, the cavity microenvironment of hollow nanomaterials upon rational design also demonstrates significant advantages in multifunctional catalysis, including bifunctional ORR/OER and OER/HER, as well as tandem catalysis. This superiority is attributed to its unique capability to achieve spatial separation of active sites and optimize mass transport conditions for multiple reactions, thereby addressing the core challenges of these catalytic systems, such as cross-reactions of intermediates, low transport efficiency of intermediates, and mismatched reaction conditions.

**Figure 18:**
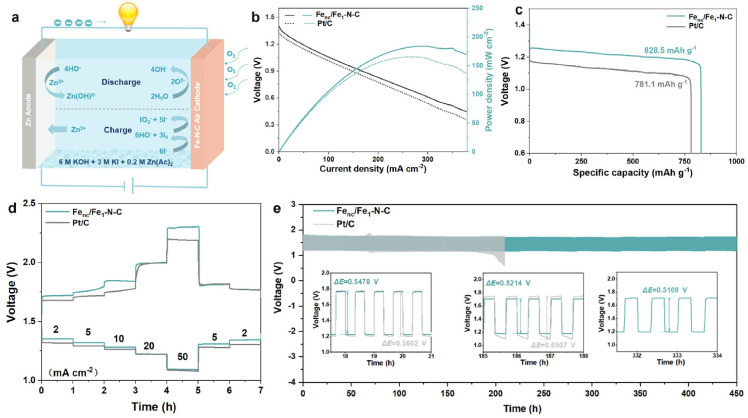
Electrochemical performance of the Zn-air/iodide hybrid batteries assembled with the FeNC/Fe_1_-N-C catalyst and the benchmark Pt/C. (**a**) Schematic diagram illustrating the reaction mechanisms during the charge and discharge processes in ZABs with KI additive. (**b**) Discharge polarization curves and the corresponding power density plots. (**c**) Specific discharge capacity measured at a constant current density of 20 mA cm^−2^. (**d**) Rate capability tested at current densities ranging from 2 to 50 mA cm^−2^. (**e**) Long-term cycling stability evaluated at 2 mA cm^−2^ [110].

Spatial compartmentalization design is the core strategy for achieving multifunctional catalysis. By rationally arranging different active sites in distinct regions of the hollow structure, including the cavity interior, outer shell, and intermediate layer, a single catalyst can catalyze multiple reactions. This strategy’s efficacy in realizing multifunctional integration is vividly manifested in the rational design of Fe cluster-liganded single-atom Fe–N–C hollow nanosheets (FeC/FeSA–HN), which fully embodies the design concept of spatial compartmentalization [110]. This hollow nanosheet architecture not only affords a high specific surface area and efficient mass/charge transport pathways but also enables the precise spatial segregation of two distinct active sites: atomically dispersed Fe-N_4_ moieties and ultrasmall Fe clusters. The synergistic interaction between these spatially separated active sites modulates their electronic structures in a coordinated manner. Specifically, the Fe clusters induce the delocalization of dz^2^ orbitals in adjacent Fe-N_4_ sites, optimizing the adsorption and desorption energetics of oxygen intermediates to achieve superior ORR performance, with a high half-wave potential of 0.931 V vs. RHE (Figure 18a–e). More importantly, the well-defined cavity microenvironment facilitated by spatial compartmentalization enables a novel tandem catalysis mechanism for hybrid batteries: the spatially isolated Fe clusters and Fe-N_4_ sites respectively catalyze the iodide oxidation reaction (IOR) and ORR, effectively replacing the destructive high-potential OER during the charging process. I^−^ is oxidized to I_2_ during charging, and the in situ generated I_2_ rapidly disproportionates into I^−^ and IO_3_^−^; subsequently, IO_3_^−^ can be efficiently reduced back to I^−^ at the bifunctional active sites in the hollow catalyst during discharging, realizing a fully closed and reversible redox cycle. This spatial and functional segregation within the hollow structure directly addresses the core challenge of mismatched reaction conditions between multiple catalytic processes, ultimately yielding a hybrid Zn-air/iodide battery with an ultralow charge/discharge voltage gap of merely 0.51 V and exceptional cycling stability over 450 h.

Another equally critical application of multifunctional hollow electrocatalysts lies in overall water splitting, which demands the simultaneous and efficient operation of both HER and OER within a single integrated system. To address this core demand and fully leverage the structural advantages of hollow nanomaterials for synergistic HER/OER catalysis, a representative study by Zhou et al. [111] vividly demonstrates a sophisticated implementation strategy, focusing on a Re and CoP hollow multi-shelled structure (Re-CoP HMSS) tailored for high-activity electrochemical water splitting. In this work, the researchers engineered a catalyst featuring a unique hollow architecture with multiple concentric shells, which inherently provides a large specific surface area, short mass/charge diffusion paths, and efficient gas release channels—key structural merits for addressing the mass transport and gas management challenges in overall water splitting. The key advancement, however, lies in the rational interfacial engineering between Re and CoP: this interface triggers significant electron redistribution, with electrons transferring from CoP to Re, thereby precisely optimizing the electronic states of both metal components. This electronic modulation synergistically weakens the hydrogen-binding energy on Co sites to enhance HER kinetics and facilitates the formation of high-valence metal intermediates that are crucial for efficient OER. As a result, this bifunctional Re-CoP HMSS achieves low overpotentials of ~107 mV for HER and ~239 mV for OER (at 10 mA cm^−2^ in 1 M KOH) and exhibits excellent long-term stability in an integrated water-splitting electrolyzer.

## 5. Conclusions and Perspectives

### 5.1. Summary of the Cavity Microenvironment Engineering Paradigm

The research paradigm of hollow nanostructured electrocatalysts has undergone a significant shift: from simply regarding hollow structures as geometric features with high specific surface area to recognizing the interior cavity as an engineerable microenvironment that directly determines catalytic performance. This review systematically constructs a comprehensive framework for cavity microenvironment engineering, which provides a unified design principle for the development of next-generation high-performance hollow electrocatalysts. The core principles summarized in this review include:

- Geometric control governs mass transport: Key geometric parameters such as cavity diameter, shell thickness, chamber structure (single/multi-chamber), and hierarchical porosity collectively construct a tunable mass transport landscape. They balance mass transport efficiency and structural stability.
- Chemical composition determines electronic structure: The electronic environment of the cavity microenvironment can be precisely regulated through heteroatom doping, internal-external surface heterojunction construction, and hydrophilic/hydrophobic surface modification. These chemical modification strategies adjust the electron density distribution of active sites, create built-in electric fields, and optimize the adsorption energy of reaction intermediates, thereby enhancing catalytic activity.
- Dynamic evolution under operating conditions: Under electrocatalytic working conditions, the cavity microenvironment undergoes dynamic changes such as surface reconstruction, and defect migration. These dynamic processes generate emergent active sites and alter the microenvironmental characteristics, so the rational design of hollow electrocatalysts must simultaneously consider both pre-designed static features and in situ evolved dynamic properties.
- Microenvironment-performance correlations provide a rational basis: By establishing the quantitative correlation between cavity microenvironmental parameters and catalytic performance metrics, this paradigm realizes the targeted optimization of hollow electrocatalysts for specific electrocatalytic reactions (ORR, OER, HER) and multifunctional catalysis scenarios.

### 5.2. Forward-Looking Perspectives and Challenges

Although remarkable progress has been made in the field of cavity microenvironment engineering for hollow electrocatalysts, there are still several critical challenges to be addressed in order to promote its practical application. At the same time, numerous promising research directions and application fields are waiting to be explored. This review summarizes the current core challenges, forward-looking research directions, and prospective application fields to guide the future development of this field.

A primary challenge is the atomic-level precision in cavity design. Currently, the synthesis of hollow nanostructures can routinely achieve nanoscale control, but sub-nanometer (angstrom-level) precision in cavity size, shell thickness, and atomic-scale distribution of heteroatom dopants remains elusive. This lack of precision limits the fine-tuning of confinement effects and electronic structures, hindering further improvements in catalytic performance. To address this issue, future research may focus on developing molecular-level templating strategies, which offer angstrom-level resolution for the atomic-scale precise construction of cavity structures. Additionally, integrating in situ characterization techniques with the synthesis process enables real-time monitoring of cavity formation, allowing feedback-adjustment of synthetic parameters to enhance precision.

Another major challenge is understanding the true operando microenvironment. Most current operando characterization techniques can only capture “snapshot” information of the cavity microenvironment at discrete time points, making it difficult to obtain continuous dynamic evolution processes. Moreover, local microenvironmental conditions within the cavity, such as local pH, potential, and concentrations of reactants and products, may deviate significantly from bulk measurement results, leading to an incomplete or biased understanding of the actual catalytic mechanism.

The need for scalability and green synthesis also poses key obstacles to practical application [112,113]. The synthesis of hollow nanostructures with complex cavity microenvironments often relies on template methods that require corrosive chemicals or high-temperature annealing processes, which are environmentally unfriendly and difficult to scale up for industrial production [114,115]. Furthermore, as structural complexity increases, batch-to-batch consistency of the prepared catalysts decreases, impeding practical deployment. In the future, developing continuous flow synthesis platforms will facilitate large-scale production of hollow nanostructures with uniform cavity microenvironments. Additionally, adopting green solvents and low-temperature synthesis routes can minimize the environmental impact of the synthesis process.

## Figures and Tables

**Table 1:** Summary of the effects of hollow nanomaterial geometric parameters on electrocatalytic performance and design guidelines [18,37,38,39,40,41,42,43,44,45,46].

Geometric Parameter	Physicochemical Impact		Electrocatalytic Implications
Cavity Diameter (D)	Diffusion path length	Smaller D shortens the mass transport path, reducing diffusion resistance; larger D extends the diffusion distance, leading to increased mass transport limitations.	Small D (<100 nm): Confinement effect is significantly enhanced, which optimizes the adsorption configuration of reaction intermediates, but strong confinement may cause limited mass transport and lower limiting current density, suitable for reactions with simple reactant/products. Medium D (100–500 nm): Achieves a balance between confinement effect and mass transport efficiency, showing universal applicability for most electrocatalytic reactions. Large D (>500 nm): Facilitates the diffusion of reactants and products, but weakens the confinement effect and structural stability, suitable for reactions involving large-molecule reactants or gas products.
	Surface curvature	Higher curvature at smaller D enhances the spatial confinement effect and induces stronger lattice strain.	
	Mechanical stability	Smaller D improves structural integrity under reaction conditions; larger D increases the risk of structural collapse due to enhanced internal stress.	
Shell Thickness (t)	Electron conduction	Thinner shells reduce the electron transfer distance, decreasing interfacial resistance; thicker shells increase the conduction barrier, impairing electron transport.	Thin shell (t < 20 nm): Enables rapid electron transfer and high active site accessibility, but poor stability limits its application in oxidative environments. Medium shell (20–100 nm): Balances electron conductivity, mechanical stability, and mass transport permeability, which is the optimal thickness range for electrocatalysts. Thick shell (t > 100 nm): Enhances structural robustness, but sacrifices conductivity and mass transport efficiency, suitable for reactions requiring long-term stability under extreme conditions.
	Mechanical rigidity	Thicker shells enhance structural stability under harsh reaction conditions (e.g., high potential, strong acid/alkali).	
	Mass transport permeability	Thinner shells promote electrolyte penetration and reactant diffusion; thicker shells form a mass transport barrier, hindering the accessibility of internal active sites.	
Single vs. Multichamber	Diffusion boundary	Multichamber structures form multiple diffusion boundaries, regulating local concentration gradients of reactants and products.	Single cavity: Features a simplified diffusion pathway, but is prone to blockage by reaction products, leading to poor long-term stability, suitable for single-step electrocatalytic reactions. Multichamber: Offers redundant mass transport channels and compartmentalized reaction zones, which not only enhance long-term stability but also enable tandem catalysis.
	Reaction compartmentalization and active site density	Multichamber configuration enables spatial separation of different reaction steps to avoid cross-reactions between intermediates, while providing additional internal surfaces for active site immobilization and increased total active site density.	
Hierarchical Porosity	Transport network construction	Macropores (>50 nm), mesopores (2–50 nm), and micropores (<2 nm) form an interconnected hierarchical transport network, realizing synergistic regulation of mass transport at multiple length scales.	Hierarchical macro-meso-micro porous structures optimize mass transport efficiency across different length scales, maximizing the utilization of internal active sites. This structure is particularly effective for reactions with complex kinetics involving multi-step electron transfer and intermediate species, as it can simultaneously accelerate reactant supply and product desorption.
	Concentration gradient formation	Porosity gradients along the shell thickness create directional diffusion pathways for reactants and products, enhancing mass transport selectivity.	

## Data Availability

Data are contained within the article.
